# “There for the safety net, not the dreams”: local decision-makers’ perceptions on scarcity, trade-offs, and caregiving responsibilities in social care resource allocation in the Netherlands

**DOI:** 10.1093/geront/gnag193

**Published:** 2026-08-12

**Authors:** Philipa Mos, Vivian Reckers-Droog

**Affiliations:** Erasmus School of Health Policy & Management, Erasmus University Rotterdam, Rotterdam, The Netherlands; Erasmus School of Health Policy & Management, Erasmus University Rotterdam, Rotterdam, The Netherlands

**Keywords:** Social care, Resource allocation, Decentralization, Prioritization

## Abstract

**Background and Objectives:**

In the Netherlands, municipalities are responsible for providing social care to enable older individuals to age in place. Resources are largely allocated by local decision-makers who translate policy into individual-level decisions, yet little is known about how they interpret and weigh relevant considerations in practice. This study examines how local decision-makers perceive the scarcity of social care resources, trade-offs they make, and challenges they encounter in everyday decision-making.

**Research Design and Methods:**

We conducted interviews with 27 local decision-makers, structured around perceptions of scarcity, trade-offs, and challenges. The analysis was guided by analytic questions focusing on interpretations of the Social Support Act, perceptions of scarcity, trade-offs, challenges in decision-making practice, and views on caregiving responsibility.

**Results:**

Local decision-makers varied in how they perceived scarcity and interpreted the statutory principles of the Social Support Act, resulting in different approaches to prioritization. Participants described a paradox between local discretion and a narrowing ability to target resources for particular priorities due to case law. Another identified challenge concerns the mismatch between policy expectations and societal views on caregiving responsibilities.

**Discussion and Implications:**

Local decision-makers’ challenges with conflicting aspects of the Social Support Act are reflected in different and sometimes incompatible prioritization approaches. Paradoxically, the Act’s broad, open formulation results in an increasingly restricted ability to allocate resources effectively in view of local needs. Better alignment between national legislation and local realities is required to allow scope for meaningful local discretion, alongside national debate to clarify priorities to support transparent and equitable allocation of social care resources.

Social care encompasses services that support individuals with long-term functional or cognitive impairments to live independently at home for as long as possible (Morris, 2004; Wiles et al., 2012). These services aim to promote self-reliance and societal participation, for example through domestic help, home adaptations, and mobility aids (Ministry of Health, 2025). In the Netherlands, the reform of the Social Support Act 2015 (Wmo 2015) marked a shift from institutional long-term care to home-based social care services, to prevent or postpone institutionalization (Maarse & Jeurissen, 2016). Since its implementation, municipalities are responsible for assessing needs and providing social care services tailored to individuals’ remaining capabilities and the availability of informal caregivers (Linthorst & Oldenhof, 2022). Beyond enabling more tailored support, this decentralization was intended to improve the efficient use of public resources by aligning service provision with local needs and priorities, and by integrating social care with other municipal services such as public health, infrastructure, and safety (Maarse & Jeurissen, 2016; Portrait et al., 2023). To this end, municipalities enjoy discretion to allocate the non-earmarked budget they receive from the national government across policy areas, including social care (Bakx et al., 2020).

Decisions on the allocation of social care resources have become increasingly important in light of growing financial pressures and broader challenges facing social care systems. Demographic aging, increased longevity, and a declining ratio of informal caregivers relative to individuals dependent on their support are driving a sustained increase in demand for social care services (OECD, 2023; The Netherlands Scientific Council for Government Policy, 2021). As public resources are finite, these pressures increasingly compel local decision-makers to make difficult choices on the allocation of social care resources and prioritize between competing demands (Clarkson et al., 2018; Mos & Reckers-Droog, 2024; Portrait et al., 2023; Stewart et al., 2021). This may result in decisions that restrict access for particular groups or withdraw specific services from public provision, which sits uneasily with the progressive realization of the right to the highest attainable standard of health. Such restrictive allocation decisions are justifiable only insofar as decision-making processes are transparent, participatory, and nondiscriminatory (UN Committee on Economic, Social, and Cultural Rights, 2007).

Yet, within the decentralized system in the Netherlands, there is variation across municipalities in how social care is organized and accessed, with patterns partly associated with municipal size and type (i.e., rural or urban) (RIVM & Cebeon, 2025). While some variation may reflect differences between local needs and context, other factors may also contribute to these differences. Earlier research on the allocation of scarce resources in social care suggests that the inherent complexity of allocation practices limits transparency (Mos & Reckers-Droog, 2024; Whitehead et al., 2026). It also indicates that informal caregivers, those with limited health literacy skills, and those with a low socioeconomic status may be disproportionately affected by the consequences of these decisions (Mackenbach, 2019; Mos & Reckers-Droog, 2026). In the specific context of decentralized social care resource allocation under the Social Support Act, research has further examined the shared perspectives of local decision-makers regarding what matters in such decisions (Mos et al., 2025). However, insight into how individual decision-makers interpret and weigh these considerations, and how such interpretations inform decision-making in practice, remains limited. The present study seeks to contribute to filling this gap.

Specifically, this study examines how local decision-makers in the Netherlands perceive and navigate the scarcity of social care resources, the trade-offs they make, and the challenges they encounter in everyday allocation decisions within the framework of the Social Support Act. While the mandate to allocate the social care budget rests with locally elected councilors, they are typically guided by appointed (i.e., non-elected) policy advisors. The latter closely collaborate with social care professionals and thus play an important role in bridging decision-making on the local and individual level. Because of this unique position, their decisions largely determine how resources are allocated and who receives support, under what conditions, and to what extent (Claessen et al., 2024; Dijkhoff, 2014; Mos et al., 2025). The decentralized social care system in the Netherlands, combined with the roles of both elected and non-elected local decision-makers, provides a distinctive context to examine how scarcity, discretion, and responsibility are navigated in practice. The results of this study are therefore relevant not only to the Netherlands, but also to other countries where social care services are decentralized and where local decision-makers exercise discretion in allocating publicly financed resources under conditions of scarcity. Moreover, insights into the interpretation and implementation of policy objectives in local practice can inform policy planning and design at the national level.

## Methods

### Sample and data collection

We conducted semi-structured key informant interviews with 27 local decision-makers with relevant expertise in social care decision-making at the municipal level in the Netherlands (Pahwa et al., 2023). Three participants were elected councilors, and twenty-four were non-elected policy advisors. Three interviews were held with two participants jointly, resulting in a total of 24 interviews conducted between May and November 2024. We anticipated that the considerations of decision-makers could vary depending on the size of a municipality, as patterns of demand for social care may differ between municipalities based on population density, population aging, and social networks. Therefore, we sampled participants purposively to include decision-makers employed in small, medium-sized, and large municipalities (defined as municipalities with <30,000 inhabitants, ≥30,000 to <80,000 inhabitants, and ≥80,000 inhabitants, respectively), and urban and rural municipalities (defined as ≥1,500 inhabitants per km^2^ and <1,000 inhabitants per km^2^, respectively) across the Netherlands (Statistics Netherlands, n.d.). We recruited participants from our professional networks, at symposia on social care, through a professional online media platform, and supplemented this with a snowball sampling approach. Table 1 below presents the characteristics of participants and municipalities.

**Table 1:** Participant and municipality characteristics.

ID	Size of municipality^a^	Type of municipality^b^	Position type
1	Large	Urban	Appointed
2	Large	Urban	Appointed
3	Large	Urban	Appointed
4	Medium	Urban	Appointed
5	Small	Urban	Appointed
6	Large	Urban	Appointed
7	Small	Rural	Appointed
8	Large	Urban	Appointed
9	Large	Urban	Appointed
10	Large	Urban	Appointed
11	Medium	Urban	Appointed
12	Medium	Urban	Appointed
13	Small	Rural	Appointed
14	Large	Urban	Elected
15	Medium	Urban	Elected
16	Medium	Rural	Appointed
17	Medium	Rural	Appointed
18	Large	Urban	Appointed
19	Large	Urban	Appointed
20	Large	Urban	Appointed
21	Medium	Rural	Appointed
22	Medium	Rural	Appointed
23	Large	Urban	Elected
24	Medium	Rural	Appointed
25	Large	Urban	Appointed
26	Medium	Rural	Appointed
27	Medium	Rural	Appointed

a Small, medium-sized, and large municipalities are defined as municipalities with <30,000 inhabitants, ≥30,000 to <80,000 inhabitants, and ≥80,000 inhabitants, respectively.

b Urban and rural municipalities are defined as ≥1,500 inhabitants per km^2^ and <1,000 inhabitants per km^2^, respectively.

Before the interviews, we obtained written informed consent from participants to record the interviews and use their anonymized data for the purpose of this study. Participants did not receive any compensation for their contribution. We do not present any detailed characteristics of the participants nor the municipality they are employed by to safeguard their anonymity. Ethical approval for this study was obtained from the Ethical Review Committee of the author’s institution (ETH2324-0640).

### Interview protocol

The interviews were conducted in-person by the first author and were preceded by an exercise in which participants independently ranked 39 statements reflecting underpinnings of social care resource allocation decisions. Statements were printed on individual cards, which participants placed on a grid ranging from “disagree most” to “agree most” according to their level of agreement. When two participants were interviewed in the same session, each participant completed the ranking exercise before the interviews commenced. The interviewer was present to provide instructions if needed, but participants completed the task independently. The results of this exercise are reported elsewhere (Mos et al., 2025). The interviews were structured around three themes aligned with the focus of this study. First, participants were asked whether and how they perceived any scarcity of social care resources within their municipality. Second, participants were invited to explain why they ranked particular statements as “disagree most” and “agree most,” in order to obtain a deeper understanding of the trade-offs and priorities underlying their allocation decisions. While the interviews focused particularly on the statements on these extreme ends of the grid, participants were invited to reflect on additional statements they considered important in this context. Third, participants were asked to reflect on challenges encountered in everyday allocation decisions. Participants were also given the opportunity to highlight any additional considerations and issues surrounding allocation decisions that had not yet been covered during the interview. The audio-recordings of the interviews were transcribed verbatim using Amberscript.

### Data analysis

We thematically analyzed the transcripts using a combined deductive and inductive approach (Braun & Clarke, 2006). Following data collection and before the analysis, we formulated a set of analytic questions focusing on interpretations of the Social Support Act, perceptions of scarcity, trade-offs, challenges in decision-making practice, and views on caregiving responsibility. These questions guided the deductive coding process and built on earlier work (Mos et al., 2025; Mos & Reckers-Droog, 2024). Although informed by the themes explored in the interviews, the analysis remained open to additional themes and insights emerging inductively from the data.

We conducted the analysis in four steps. First, the first author imported the transcripts into ATLAS.ti25. Second, following familiarization with the data, the first author coded all transcripts using deductive and inductive codes and developed a preliminary framework comprising themes and categories. Third, to refine and validate the framework, the second author independently coded five transcripts. Differences were discussed and resolved through consensus, resulting in revisions to the coding framework. The second author subsequently coded an additional five transcripts to assess whether the identified themes and categories adequately captured the data and whether the reporting of findings accurately reflected the interviews. Minor refinements were made to both the framework and the presentation of results. Finally, the first author conducted a second round of coding across all transcripts to apply the revised framework consistently and further refine the themes and categories.

## Results

Table 1 presents the characteristics of the participants and municipalities. The 27 participants were employed in one of 15 different municipalities in the Netherlands; 3 were employed in a small, 11 in a medium-sized, and another 11 in a large municipality. Furthermore, 16 worked in an urban and 9 in a rural municipality.

In line with the aim and methodological approach of this study, three themes were developed deductively: (A) Perceptions on scarcity within the municipality, (B) Trade-offs and priorities underlying allocation decisions, (C) Challenges encountered in everyday allocation decision-making. In addition, one theme was developed inductively: (D) Solidarity and shared caregiving responsibilities. In what follows below, we narratively report on the results from the interviews structured by these themes. Verbatim quotes of participants are presented in italics and indicated by participants’ identification number (ID). Note that “participant” denotes both elected and non-elected decision-makers; distinctions between these are made when relevant.

### Theme A: Perceptions on scarcity within the municipality

Participants expressed divergent perceptions of the scarcity of social care resources. Pressure on the system was frequently linked to a growing gap between demand and financial and workforce capacity, demographic aging, and policy changes. Participants particularly referred to the introduction of the flat subscription rate (of €19 per month in 2025; Vereniging Nederlandse Gemeenten, n.d.) for social care in 2019, which substantially lowered user charges for many households. As one participant described, “*this had a magnetic effect on [the demand for] domestic support*” (ID21). While some participants described financial pressure as structural, others perceived scarcity as a more recent development following periods of relatively abundant funding and higher expenditure.

In contrast, others suggested that concerns about scarcity in social care were overstated: “*we can do with ten percent less*” (ID8); that it is a matter of framing rather than a structural constraint; or stipulated that scarcity does not exist in social care because of the open-ended budget following the legal obligation to provide support to those with recognized needs. As one participant explained: “*If the [social care] budget is depleted, we’ll just have to make money […] we must provide [it] regardless*” (ID5). Financial pressures are thus absorbed by reallocating resources from other municipal departments and perceived as the need to “*make other choices*” (ID10) rather than absolute scarcity. This reflects that social care is prioritized relative to other public services, such as infrastructure, which was made explicit by several participants who mentioned that “*[social] care and support are our top priorities [...] I don’t think we can escape that*” (ID27); “*these are vulnerable individuals, and they have to be supported*” (ID6), and “*if it is necessary, we must provide it, and if that means we exceed the budget, than that’s just the way it is*” (ID13).

In line with this, participants emphasized that allocation decisions were primarily about fulfilling their responsibility to provide support and not about saving money: “*we should ensure that individuals receive what they need […] then we consider whether it can be provided in a less costly way*” (ID23). In addition to this, some highlighted the long-term benefits of social care, although these are not immediately reflected in (short-term) financial accounts. At the same time, other participants cautioned that reallocating resources away from other services such as libraries, sports facilities, and public spaces could ultimately undermine social care objectives, as “*some things are also part of the solution, you should certainly not cut back on those*” (ID16).

Despite differences in how participants perceived scarcity, participants converged in identifying structural pressures, particularly those related to increasing demand, workforce shortages, decreasing availability of informal caregivers, and constrained resources. As one participant noted, “*the bigger problem is that providers are far from available on every street corner*” (ID1). Maintaining service provision under these conditions was perceived as increasingly difficult, with one participant noting that “*our social care system, as it currently operates, is unsustainable, unaffordable, and will not endure*” (ID14). Yet, responsibility for making difficult allocation decisions was often deflected. Policy advisors discussed potential solutions to foster the financial sustainability of the social care system, while emphasizing that final decisions rest with elected councilors. Participants acknowledged that difficult allocation decisions may be politically sensitive and therefore put off but also criticized the lack of political will to make them: “*Frankly, I would give the councilors a failing grade*” (ID6). Another participant noted that “*politicians never like making sharp choices, […], it’s more appealing to cut a ribbon*” (ID14). Notably, these participants suggested that reductions in service levels may be more readily accepted if necessitated by workforce shortages rather than by financial constraints.

In sum, participants discussed structural pressures arising from increasing demand, workforce shortages, and constrained resources when reflecting on resource scarcity in their municipalities. While some experienced these pressures as constituting scarcity, others described scarcity as a frame rather than reality. The accounts of participants did not differ by size or type of municipality.

### Theme B: Trade-offs and priorities underlying allocation decisions

Throughout the interviews, participants described multiple, partly overlapping priorities in allocation decisions under conditions of scarcity. Central among these was needs-based prioritization, which was operationalized in different ways, including the pursuit of equal opportunities, broad service coverage, and an emphasis on prevention and enablement.

Nearly all participants noted that municipalities have an obligation to provide services to those in need, suggesting that resource allocation should largely follow demand. At the same time, many emphasized that individuals with the greatest need should be prioritized. This implied that further prioritization may be required within this group. Nonetheless, who constitutes this group of “individuals with the greatest need” remained difficult to specify. Rather than a clearly operationalizable allocation principle, the concept appeared to reflect a political or normative stance that participants themselves struggled to articulate. As one participant noted: “*Those who truly depend on support should come first. But [deciding whether] someone is truly dependent is something we can certainly disagree on*.” (ID14).

Participants described several factors in determining need, including one’s (dis)abilities, urgency, and the expected duration and foreseeability of need. Severe impairments were commonly perceived as strengthening claims to public support, whereas milder impairments were associated with a greater expectation of self-management. Urgency was also mentioned as an important determinant, though sometimes difficult to assess. As regards the expected duration and foreseeability of need, participants felt that those with temporary or predictable limitations, such as recovery from planned surgery, could be expected to arrange support privately in advance. Moreover, one participant noted that requests for housing adaptations could be denied when individuals had foreseeable limitations but did not anticipate these when moving to a new home. Assessments of need were also shaped by individuals’ own resources, particularly the availability of informal support and financial means. One participant noted: “*We take a step back in the case of individuals who are able to contribute more themselves.*” (ID27).

A second priority was promoting equal opportunities and ensuring a basic standard of living, often referred to by participants as achieving “a six.” This objective reflects a specification of the needs-based approach, in which support is aimed at bringing individuals up to a sufficient standard. Participants described this threshold as the ability to maintain a clean home and sustain social contacts. One participant explained: “*If you can get yourself to a five, we’ll help you to go from there to a six. If you can only get yourself to a three, we’ll take responsibility for getting you from three to six. It is okay to differentiate between individuals [in that manner].*” (ID7). Anything beyond “a six” was viewed as outside the municipality’s responsibility. As one participant noted: “*We are there for the safety net, not for the dreams*.” (ID15). Participants described tailoring support to individuals’ capacity to maintain this minimum independently, in line with the emphasis on personalization in the Social Support Act. This involved identifying underlying needs through dialogue. In tandem, participants highlighted individuals’ tendency to compare their support with that received by others and heightened expectations, sometimes going beyond what municipalities considered necessary to maintain the minimum standard. This could result in conflicts with individuals.

A third priority emphasized maintaining broad coverage, based on the obligation to provide support to all individuals with a recognized need. Several participants believed that the Social Support Act leaves little room to prioritize between individuals and that instead, they had to find ways to provide support to as many individuals as possible: “*As a pater familias, you have to see how we can help as many people as possible with the available money*.” (ID10). This view was grounded in the assumption that individuals generally request support only when necessary: “*No one applies for support if they don’t need it*.” (ID22). Participants believed that individuals first seek support in their network and therefore felt that requests for formal support should be taken seriously. Notably, this reflected a more positive view of individuals’ behavior and motivations when asking for public support compared to other accounts. To sustain broad coverage, participants described various strategies, including reducing support levels, applying stricter assessments, and increasing user contributions. Many also referred to investing in collective provisions that do not require prior needs assessment. These were described as promoting social networks and reducing isolation. Some participants noted that collective provisions may involve greater reliance on informal support. Moreover, broad coverage may come at the detriment of support quality. While aiming to maximize aggregate rather than individual wellbeing, participants acknowledged the limits of this approach: “*Support levels can certainly be reduced, but we are wary of hitting a bottom line. That is the dilemma, the conflict that we are currently facing*” (ID26). Participants also acknowledged that tailored support may still be necessary when collective provisions are insufficient. One participant noted: “*It has to yield something. Providing someone with something that is of no use is a waste*.” (ID10).

Finally, participants emphasized prevention and supporting individuals’ self-reliance and participation as a priority. Participants described prevention as proving timely support to avoid deterioration. One participant explained: “*Targeting people who, if you don’t help them, end up in a downward spiral, drifting away from society*.” (ID8). Supporting individuals early, while they remain at home, was seen as consistent with both individual preferences and system sustainability. Another participant noted: “*People thrive when they are independent in their own environment.*” (ID15). Participants described prevention as extending beyond social care to activities that support community engagement. Some referred to collective provisions as enhancing social cohesion and accessibility. One participant noted: “*Provisions on a walk-in basis enhance cohesion and social interaction. It is much cheaper, and also preventive, we’re encouraging the community to look out for each other*.” (ID13). Some reported that their municipality had abolished user fees for collective provisions in order to improve accessibility.

Taken together, these priorities illustrate how participants navigate the allocation of limited resources in the face of growing demand. Although presented separately, these priorities are not necessarily mutually exclusive. The finding that participants discuss multiple priorities may signal reluctance to make explicit trade-offs between them.

### Theme C: Challenges encountered in everyday allocation decision-making

Participants described several challenges in everyday allocation decision-making. These included limited municipal discretion under the Social Support Act, high public expectations, and difficulties in coordinating responsibilities across the health, social, and long-term care systems.

Although municipalities were promised greater discretion over the local organization of social care and related allocation decisions, participants described national policies and case law aimed at safeguarding the rights of vulnerable individuals as restricting municipal control over access to services. As one participant put it: “*The premise of decentralization, giving municipalities more freedom, has failed completely*” (ID1). Another noted: “*The legislation is so broad, saying that we have to do everything one asks for, that we as a municipality find it hard to set limits*” (ID19). Municipalities’ scope to determine the forms of support and to define what needs warrant public support is limited. Instead, municipalities must provide support to individuals with recognized needs, limiting their ability to allocate resources effectively. One participant remarked: “*Sometimes, social care feels a bit like a deep black hole […] it has to be paid for, and budgets have to grow accordingly.*” (ID18). Notably, some participants accepted these constraints, while sought the boundaries of their discretion, for instance by discouraging wealthier individuals from applying for support: “*We want to allocate the scarce resources fairly, and we prefer to distribute them among financially vulnerable individuals.*” (ID5).

As a result of the inability to specify the scope of the social care system at the local level, service provision may be determined by what individuals ask for, rather than their objective needs. In this respect, participants described perceptions of entitlement and “shopping behavior” among individuals. One participant noted: “*People need to a change their mentality; the municipality is not an ATM where you insert a card and specify what support you want*” (ID14). Participants further described expectations as shaped by social norms: individuals may struggle to accept that public support cannot meet their former standard of living nor is available for any problem at any time. Participants associated these tensions with the shift toward collective provisions. Moreover, participants observed that individuals with higher levels of health literacy may be more successful in obtaining support, sometimes by directly approaching councilors or policy advisors. One participant noted: “*Not everyone benefits equally from social care in the Netherlands. We support people not because they have imminent needs, but because they know how to navigate the system. I think that’s an unfortunate development.*” (ID19).

Participants also described challenges related to coordination between public health, long-term care, and social care. Some characterized social care as a last resort for individuals who fall outside of the scope of other systems: “*It has become the dustbin [of the health care system]*” (ID13). Participants described transitions from social care to long-term care as challenging. It is fraught with discontinuity, complex procedures, and limited long-term care capacity. They noted that individuals often hesitate to make that transition because they are attached to familiar routines and professionals, and application procedures for long-term care are complex. Participants also reported that limited long-term care capacity means that only severe cases are admitted. Although such cases formally fall outside the scope of social care, participants described situations in which they continued providing support due to established involvement with the individual. Participants further described a sense of responsibility to prevent individuals from falling through systemic gaps and reported encountering the practical consequences of policy decisions for individuals in their daily work.

Overall, local decision-makers described several institutional and practical challenges in carrying out their statutory responsibilities under the Social Support Act. Legal constraints restrict local discretion over access to social care, meaning that service provision is influenced not merely by assessed needs but also by individuals’ demands. This may contribute to inequities in access to services, as this may depend on individuals’ ability to articulate and advocate for their subjective needs. In addition, participants highlighted difficulties in coordinating with other parts of the care system, and in bridging gaps in service provision.

### Theme D: Solidarity and shared caregiving responsibilities

Participants described how allocation decisions in social care are closely tied to views on solidarity and caregiving responsibilities, discussing how responsibility for support is shared between the state, individuals, and society, and how this relates to perceptions of disability and illness.

Aside from the legal obligation to provide support to individuals with recognized needs, some participants also expressed a strong sense of solidarity with individuals with (severe) disabilities. These participants described prioritizing services or devices that individuals cannot easily arrange themselves, such as those that are medical, highly specialized, or expensive. They considered user fees unfair in these situations, as they feared discouraging individuals from seeking necessary support. This contrasted with the widely shared view that user fees for domestic support should be raised, as such services were often described as everyday forms of support that individuals could reasonably arrange themselves. Several participants described aims to normalize such forms of support and to discontinue their public provision of those. Participants mentioned that electric bicycles and raised toilet seats had been redefined in this way.

Participants noted that age- and disability-related limitations have become overly medicalized and have resulted in increased expectations of the scope and extent of public support. As one participant remarked: “*Decline is part of life, and it is not a task of government to provide expensive facilities to individuals simply because they insist on a certain living standard.*” (ID14). Another noted that “*we also don’t give out buggies for infants*” (ID9). In particular, when individuals have the resources to arrange support themselves, participants believed that public resources should be reserved for “*people who cannot take control, do not have the salary or the resources, who do not have the network*” (ID7). A considerable number of participants discussed user contributions and wished to consider income or wealth in needs assessment, even though municipalities are legally restricted from inquire about income or wealth. Some participants associated recent policy changes, abolishing income-based differentiation for domestic support, with increased demand among wealthier individuals.

Motivated by concerns about fairness, particularly when individuals with sufficient resources demanded domestic help and housing adaptations, some municipalities used stricter prioritization practices. Participants were concerned that demand among higher-income groups impacted the capacity to support individuals with little alternatives to public support. As such, participants described using informal strategies to prompt individuals to rely on informal care and reconsider their request for formal support. These approaches included conversational strategies, encouraging individuals to seek support from informal networks, and waiting list prioritization that implicitly considered whether individuals arranged private support while waiting. One participant stated: “*People with lower incomes end up on waiting lists because those who can easily arrange it themselves ask for support. I find that very unfair.*” (ID5).

Societal caregiving responsibilities were primarily described in terms of informal care. While participants stressed the importance of informal caregivers due to limitations of the formal care system and the social benefits of informal caregiving for both recipients and caregivers, they could not oblige relatives or volunteers to provide support. Participants also referred to cultural and social barriers, including deep-rooted traditions of individualism. As one participant noted: “*In the Netherlands, we’re individualistic and everyone only cares for themselves*” (ID16). Nonetheless, some participants described attempts to encourage informal care by narrowing the scope of formal services. Others suggested that mobilizing informal caregivers and volunteers may be more feasible in smaller municipalities with stronger community ties. They also recognized caregiver burden, and several participants spoke from personal experiences with informal caregiving. One noted the difficulty of combining caregiving responsibilities with employment: “*Informal care is a scarce commodity, we have to cherish it. If the burden becomes too heavy, it may cause people to drop out of work, and you get a kind of waterbed effect.*” (ID7).

In sum, the allocation of social care resources on the local level is inextricably linked to views on solidarity and the distribution of care responsibilities between municipalities, individuals, and informal networks. Participants described tensions between encouraging self-reliance and informal care while ensuring access to publicly funded support for individuals with the greatest needs.

## Discussion

The implementation of the Social Support Act in 2015 marked a shift in the organization of support for individuals with long-term disabilities and limited self-reliance, particularly older individuals, in the Netherlands, moving responsibility for service provision and resource allocation to the municipal level. The Act reflects a broader policy shift toward greater reliance on informal care and reduced dependence on centrally organized public provision of social care. Although this shift was expected to contain public spending, demand for social care remains high, reinforcing the need for prioritization in allocation of limited resources (Ministry of Health, 2025). The aim of this study was to examine how local decision-makers perceive and navigate the scarcity of social care resources, the trade-offs they make, and the challenges they encounter in everyday decision-making. Based on interviews with 27 local decision-makers, we identified four themes capturing local decision-makers’ (A) perceptions on scarcity within the municipality, (B) trade-offs and priorities underlying allocation decisions, (C) challenges encountered in everyday allocation decision-making, and (D) solidarity and shared caregiving responsibilities.

While local decision-makers may not universally label it as scarcity, our findings of the priorities and trade-offs they make in allocation decisions suggest they are limited in their ability to meet demand. Findings regarding Theme B illustrate that decision-makers uphold four different priorities when mediating demand with available resources. Each priority reflects different interpretations of the statutory principles of the Social Support Act. Needs‑based prioritization and broad coverage correspond with the statutory obligation to support individuals with recognized needs. The pursuit of equal opportunities reflects the Act’s emphasis on personalization, while the focus on prevention and enablement aligns with the policy objective of avoiding or delaying institutionalization and strengthening community bonds. Notably, participants’ responses sometimes implied they uphold multiple priorities. Although interconnected, attempting to fulfill multiple priorities simultaneously may ultimately result in prioritizing nothing.

What could be described as “priority-setting paralysis” appears rooted in the Social Support Act’s broad and open nature. Findings from Theme C highlight that participants grapple with friction between their local discretion and legal constraints. Although the Act grants participants discretionary room, municipal control over access is limited, as social care provision is a legal obligation and support cannot be denied solely on the basis of scarcity. Moreover, the open wording of the Act is increasingly narrowed by case law, establishing standards that municipalities must uphold universally. While these developments aim to safeguard legal certainty and protect vulnerable groups (Ministry of Health, 2025), this corrective mechanism runs counter to one of the Act’s main features, namely local discretion, limiting local decision-makers’ ability to allocate resources effectively.

Findings from Theme D suggest that informal care is less engrained in society than policymakers envisioned when introducing the Social Support Act 2015. This mismatch between policy assumptions and actual support-seeking behavior may create friction for local decision-makers. Participants often expressed critical views toward individuals seeking public support, particularly when they believed support could be arranged privately. This finding aligns with those of earlier research showing that decision-makers in the Netherlands may blame citizens for the failure of public service provisions (Verhoeven & Tonkens, 2013). During the interviews, individuals were often portrayed as wanting more than was considered necessary, illustrating how expectations of local decision-makers may not accord with how individuals and their networks behave in practice.

Our findings shed light on the translation of national policy into local contexts through local allocation decisions. Differing interpretations of the Social Support Act give rise to different allocation practices, which in turn shape individuals’ access to social care and may contribute to variation in the organization and functioning of local social care systems. This study provides unique empirical insight into how local decision-makers in the Netherlands, who operate largely out of public view, interpret and weigh considerations in decisions on allocation. Our sample included local decision-makers from 15 municipalities in the Netherlands. Although further research is needed to assess the extent to which our findings are generalizable to all local decision-makers and to identify patterns related to municipal size and type, the context of financial and workforce shortages in which they operate is likely similar to that of other municipalities. Moreover, while our study was conducted in the context of decentralized decision-making in the Netherlands, we believe our findings are relevant to other settings where local or regional authorities interpret and implement nationally defined policy frameworks, illustrating how local allocation decisions serve as a mechanism through which national policy shapes local social care systems.

Therefore, we believe our findings have important implications for policymakers. First, national decision-makers could better account for local realities and entrenched cultural traditions, including the limited support for informal caregiving. This could involve reassessing assumptions about the role of informal care in needs assessments and aligning policy expectations with empirical evidence on care practices. Second, the combination of conflicting legal objectives, limited formal guidance and constrained discretion may prompt local decision-makers to adopt allocation practices that diverge from (some of) the intentions of the Social Support Act 2015. Such tensions may increase the influence of personal considerations in decisions while simultaneously limiting scope for innovation. National decision-makers could improve internal coherence of the Act’s objectives and provide clearer guidance on how to balance competing aims on the local level. This could include the development of a decision framework that makes explicit how trade-offs can be handled in practice. Third, the legitimacy and acceptability of local allocation decisions depend on transparency and participatory processes. Involving citizens and social care recipients in the design and evaluation of policies can help local decision-makers better capture experiences and preferences of those that are impacted by the policies and enhance fairness and acceptability.

Participatory processes may furthermore increase the transparency of allocation decisions. All in all, our findings signal the need to discuss what should receive priority in allocation decisions on social care. Although states, including municipalities, must take progressive steps to realize the right to the highest attainable standard of health and wellbeing to the maximum of their available resources, restrictive allocation of public resources may be permitted if financial scarcity necessitates it. However, our findings that local decision-makers often do not discuss scarcity explicitly and uphold multiple prioritization approaches indicate potential nonconformity with the requirement of these decisions to be transparent, participatory, and nondiscriminatory in their effects (UN Committee on Economic, Social, and Cultural Rights, 2007). We argue that failing to explicate prioritization approaches may undermine the equitable distribution of available resources and comes at the expense of future support needs and other public goods (Mos & Reckers-Droog, 2026).

There are some limitations to our study that are worth noting. First, we relied on self-reported accounts of allocation practices, and we cannot guarantee whether these fully reflect participants’ actual decisions. Future research using data triangulation may provide additional insight into how the identified considerations translate into decision-making practice. Second, given that the social care budget is open-ended, allocation decisions cannot be seen separately from resource allocation to other public services. Follow-up research is necessary to examine how local decision-makers balance social care against other competing priorities, and the consequences of this. Moreover, while findings of our study reveal inconsistencies between national policy objectives and local implementation, future research is warranted to explore how to mediate resource scarcity and local discretion without undermining legal certainty.

In conclusion, local decision-makers were given responsibility and discretion in 2015 to allocate social care resources in the Netherlands, yet our findings highlight that their ability to do so effectively is limited. Indeed, they must uphold the objectives and ideology underpinning the Social Support Act while being confronted with financial and workforce shortages, constrained discretion, and cultural norms regarding caregiving. Their ability to allocate resources to particular groups often conflicts with the safeguarding of the rights of individuals. Despite efforts to enhance legal certainty for vulnerable groups, the compromises local decision-makers have to make may still result in an inequitable distribution of resources, potentially favoring individuals with strong health-literacy skills. To ensure that scarce social care resources are allocated fairly and without unintended discrimination, it is imperative to develop national guidelines that make explicit how prioritization should be handled in practice.

## Data Availability

To protect the privacy of the study participants, the authors are unable to publish the data used for this study to be made available to others.
